# Hypovitaminosis D and Its Associated Factors in North Algerian Postmenopausal Women: Results of a Cross-Sectional Study

**DOI:** 10.1155/2017/9032141

**Published:** 2017-12-17

**Authors:** S. Oussedik-Lehtihet, C. Haouichat, N. Hammoumraoui, E. Ducros, C. Gouhier-Kodas, S. Lancrenon, H. Djoudi

**Affiliations:** ^1^Centre Hospitalier Universitaire de Douera, Rue des Frères Halim, Douera 16000, Alger, Algeria; ^2^Laboratoire Innotech International, 22 Avenue Aristide Briand, 94110 Arcueil, France; ^3^Sylia-Stat, 10 Boulevard du Maréchal Joffre, 92340 Bourg-la-Reine, France

## Abstract

**Purpose:**

As the vitamin D status of Algerian postmenopausal women was poorly described, this cross-sectional study investigated the prevalence of low vitamin D status in a sample population. Secondarily, predictive factors of this hypovitaminosis D were explored.

**Methods:**

All the 336 selected women ≥ 45 years from Douera were interviewed to get anthropometric and lifestyle data, reproductive and medical history, medications, and calcium/vitamin D intakes. A blood sample was collected to measure 25-hydroxyvitamin D (25(OH)D) concentrations.

**Results:**

Approximately 86% of subjects had low vitamin D status (<20 ng/mL). Mean 25(OH)D level was 14.4 ± 5.3 ng/mL with a clear seasonal dynamic and a significant negative correlation with PTH levels (*r*  = −0.15, *p*=0.006). A multiple regression analysis using the 25(OH)D cutoff value of 17 ng/mL instead of the generally admitted level of 20 ng/mL was performed to increase statistical power. Other seasons than summer (OR 4.159 and 95% CI 2.456–7.043), obesity (≥30 kg/m^2^, OR 1.826, 95% CI 1.081–3.083), and veiling (OR 3.526, 95% CI 1.090–11.400) were significantly associated with 25(OH)D concentrations <17 ng/mL.

**Conclusions:**

In North Algeria, the abundant sunlight appears insufficient to fully offset hypovitaminosis D risk factors in postmenopausal women, especially obesity and veiling. It suggests the major need to increase vitamin D supplementation in this subpopulation.

## 1. Introduction

Vitamin D inadequacy is highly prevalent worldwide [1] and has well-known direct and deleterious consequences on bone health. This mainly occurs via secondary hyperparathyroidism, leading to excessive bone remodelling and ultimately to bone weakening, which in turn increases fragility fracture risk. Vitamin D deficiency leads to rickets in children and osteomalacia in adults, and long-term insufficiency contributes to osteoporosis [2, 3]. Serum circulating 25-hydroxyvitamin D (25(OH)D) level is considered as the best indicator of the body vitamin D status, and although still debated, most experts defined vitamin D deficiency as 25(OH)D levels lower than 20 ng/mL [4, 5].

Despite the fact that all age groups may be affected, it became evident that vitamin D inadequacy is particularly common among older adults, which is of substantial concern in the context of the significant increase in ageing populations and life expectancies worldwide. Lack of exposure to sunlight, which is the primary source of vitamin D, poor nutrition, and a decrease in the capacity to produce vitamin D_3_ in the skin are the main hypothesized causative factors of vitamin D inadequacy in this subpopulation [6]. Hence, not surprisingly, postmenopausal women from different countries were reported to be particularly vulnerable to vitamin D deficiency, which leads numerous international health authorities and medical societies to produce recommendations for the management of postmenopausal osteoporosis by maintaining adequate vitamin D levels by supplementation.

However, data obtained from various populations of community-living postmenopausal women with or without osteoporosis highlight a wide disparity of prevalence rates of 25(OH)D concentration ≤ 20 ng/mL depending on the considered geographical areas [7, 8]. For instance, in a European study involving more than 8500 postmenopausal women, the prevalence of 25(OH)D deficiency reached 32.1% when considering the cutoff value of 20 ng/mL [9], while in Indian, Chinese, and US populations of postmenopausal women, the estimated prevalence rates were 53.3%, 72.1%, and 53%, respectively [10–12].

Data related to the vitamin D status of postmenopausal women in North Africa are scanty or lacking, and to our knowledge, such information has not been documented in Algeria. Located in the Mediterranean basin, Algeria is characterized by a climate that is mostly sunny around the year. Consequently, since vitamin D status is most strongly influenced by skin synthesis resulting from exposure to UVB (sunlight), a proper amount of vitamin D is theoretically to be expected in Algerian postmenopausal women. Nevertheless, there is accumulating evidence of the frequent occurrence of subclinical vitamin D deficiency in populations living in sunny countries [13–17]. Moreover, vitamin D insufficiency was recently found to be frequent in healthy Algerian children [18].

Hence, although extrapolating the prevalence data from countries of similar climates and latitudes should be cautious because of other influencing factors like diet on vitamin D status, we hypothesized that Algerian postmenopausal women may not be an exception and that vitamin D inadequacy may be relatively prevalent in this age group. To provide an estimation of the magnitude of the problem in our country, a cross-sectional study was carried out to determine the vitamin D status among a healthy postmenopausal women population recruited in Douera, a city located in the extreme northern part of Algeria. The secondary objective was to identify the determinants of hypovitaminosis D in order to identify the most vulnerable patients on whom public health actions should be primary focused.

## 2. Methods

### 2.1. Study Design and Population

This cross-sectional prospective study was conducted between April 2008 and November 2009 in Douera, a city of approximately 57,000 inhabitants located in the district of Algiers (Algeria) at a latitude of 36°40′ North and whose demographic characteristics, notably in terms of age and gender distribution, are representative of those of the whole Algerian population (*Office National des Statistiques*, Algeria, http://www.ons.dz). Participants were recruited by medical students according to a traditional and recognized door-to-door sampling method. After a short interview using a structured questionnaire in their homes, the eligible female patients were invited to participate in the study and to attend the Specialized Hospital Establishment (EHS) of Douera. All volunteer participants were provided with full information about the study's purpose, and they gave informed consent to participate in the study. The study was approved by the ethics committee of the hospital.

Interviewed women had to meet the following inclusion criteria: age 45 years or older, living in Douera, and being postmenopausal. The main noninclusion criteria were any musculoskeletal, thyroid, hepatic, or renal diseases, granulomatous diseases, sequels of extensive burn injuries, or intake of any medication that may impact vitamin D status or bone metabolism (e.g., glucocorticoids and anticonvulsants), including calcium and/or vitamin D supplementation and multivitamin supplementation during the three preceding months. Additionally, patients who had been treated with osteoporotic medications (e.g., hormonal replacement therapy, selective estrogen receptor modulators, and bisphosphonates) within the previous 6 months were not recruited into the study.

### 2.2. Clinical and Sociodemographic Characteristics

After the participants signed the informed consent, they underwent a clinical examination. The clinical data collected included age, height, and weight. Body mass index (BMI) was then calculated using these anthropometric data as body weight in kilograms divided by height in meter squared. Medical (e.g., existence of chronic illness) and obstetrical (parity) histories, age at menopause, current medications, and socioeconomic status (education level and profession) were also obtained by an experienced investigator. Participants were also asked to describe their previous fractures, if any (number and site).

On the same day, bone mineral density (BMD) at L1–L4 lumbar spine and total hip, expressed in grams per centimeter squared, was measured in all participating women by dual energy X-ray absorptiometry (DXA–QDR-2000/W, Hologic Inc.). Daily quality control was performed by phantom measurements (DPA/QDR-1 anthropomorphic spine phantom). At the time of the study, phantom measurements showed stable results. The phantom precision expressed as the coefficient of variation percentage was 3%. T-scores of the lumbar spine and total hip were calculated using the reference curve obtained with the French OFELY cohort [19], preferred to the US NHANES reference curve in the absence of an Algerian one, and both were previously shown to give comparable results [20]. The World Health Organization classification system was applied, defining osteoporosis as T-score ≤ −2.5 and osteopenia as T-score between −2.5 and −1 [21].

### 2.3. Questionnaires

A series of questionnaires were used for data collection. The skin phototype was determined according to the Fitzpatrick classification from I (pale white skin) to VI (dark brown or black skin). Daily sun exposure (average duration and sun-exposed body areas) and dressing habits were evaluated by direct questioning. Similarly, the mean weekly vitamin D intake from food was assessed using a six-question tool [22, 23]. The daily calcium intake was calculated using the validated self-questionnaire of Fardellone and coworkers [24], adapted to the Algerian eating habits.

### 2.4. Laboratory Tests

A venous blood sample was collected on the same day of bone density measurement for the determination of 25-hydroxyvitamin D (25(OH)D) and parathyroid hormone (PTH) serum concentrations by electrochemiluminescence immunoassay using the Roche Elecsys 2010 COBAS system. This method can measure the concentration of 25(OH)D in the range of 10–250 nmol/L, and PTH in the range of 1.2–5000 pg/mL. All laboratory tests were performed at the Specialized Hospital Establishment (EHS) of Douera.

### 2.5. Statistical Analysis

Analyses were performed with SAS software (version 9.4, SAS Institute Inc., Cary, NC), and statistical significance was defined as a value of *p* < 0.05.

To compare two groups, we used the chi-square test for categorical variables expressed as frequencies, the *t*-test for continuous variables expressed as mean ± standard deviation (SD), and the nonparametric Mann–Whitney test when the assumption of normality was questionable. To compare more than two groups, we used the chi-square test for categorical variables, a one-way analysis of variance for continuous variables, and the nonparametric Kruskal–Wallis test when the assumption of normality was questionable. Pearson's correlation was used to study the relationship between quantitative variables.

A multiple logistic regression analysis was used to identify the factors best explaining a lower level of 25(OH)D. A stepwise procedure was used to select variables. Stepping was stopped when there were no further candidate variables that would enter the model at the 5% significance level.

## 3. Results

A total of 400 women were contacted, interviewed, and screened. Less than 10% of the screened participants did not attend the EHS of Douera to undergo the clinical examination. Among the participants who were attending the hospital, some presented with noninclusion criteria and were excluded (currently with thyroid disease, treatment with osteoporotic medications within the previous 6 months, or treatment with calcium and/or vitamin D supplements during the three preceding months).

### 3.1. Sociodemographic and Clinical Characteristics of the Population

The study involved 336 women, mainly without any education level (57.7%), residing in the urban areas of Douera (74.1%), and aged between 45 and 87 years (Table 1). The recruitment of participants was well balanced over the seasons. The average parity was 6.5 children per woman, and the mean age at menopause was 47.4 years. More than half of the study participants had skin phototypes I–III (50.6%), while 49.5% had skin phototypes IV-V. Almost all women (96.1%) wore a veil covering their hair, arms, and legs. The mean BMI was 28.51 ± 5.32 kg/m^2^. One hundred and forty subjects (42.6%) were obese (BMI ≥ 30 kg/m^2^), while 117 (35.6%) were overweight (25 kg/m^2^ ≤ BMI < 30 kg/m^2^).

The participants did not take any calcium nor vitamin D supplements within the three months prior to the study. The daily dietary calcium intake was estimated to be less than 500 mg/day in 58.3% of the participating women, which is much lower than the recommended daily allowance ranging from 1000 to 1200 mg/day in this population [4]. On the basis of a questionnaire, the mean daily vitamin D intake from dietary sources was estimated to be 1.4 ± 1.1 µg/day (56 ± 44 IU/day). Self-report on sun exposure of subjects was not analyzed because most of the women did not report or report only very limited exposure to the sun, even in their garden or courtyards, for example. The diagnosis of osteoporosis was confirmed by a T-score of hip and/or lumbar spine ≤ −2.5 in 32.8% of the participants. Based on self-report history, the number of subjects with a history of at least one fracture was 54 (16.1%). The most frequent site of the fracture was the wrist (*n* = 15). Other fractures were located in the lower (*n* = 22) or upper (*n* = 9) parts or were thoracic fractures (*n* = 3). Three participants could not remember the exact localization of their previous fracture.

### 3.2. Serum 25(OH)D and Prevalence of Hypovitaminosis D

**Figure 1:**
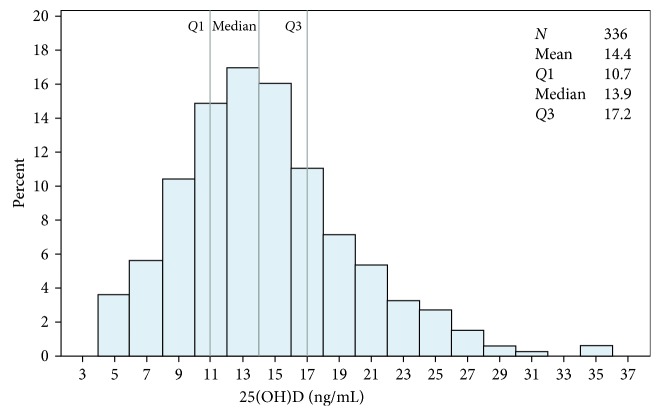
Distribution of the 25-hydroxyvitamin D (25(OH)D) concentrations across the entire study population (*n* = 336).

The mean serum concentration of 25(OH)D for all 336 subjects was 14.4 ± 5.3 ng/mL. The prevalence of circulating 25(OH)D levels less than 20 ng/mL was 85.7%. The 25th and 75th percentiles of the distribution of 25(OH)D concentrations were 10.7 and 17.2 ng/mL, respectively. The distribution of subjects across ranges of 25(OH)D concentrations is shown in Figure 1. A seasonal dynamic in 25(OH)D levels was described with a peak in the summer months versus the other three pooled seasons (17.2 ± 6.2 versus 13.4 ± 4.7 ng/mL, *p* < 0.0001, Mann–Whitney test). It should be noted that the different seasons were determined according to local criteria: winter covers December, January, and February; spring covers March, April, and May; summer covers June, July, and August; and autumn covers September, October, and November.

A significant negative correlation was observed between PTH and 25(OH)D levels (Pearson's correlation coefficient = −0.15186, *p*=0.006). The mean PTH concentration was 64.2 ± 25.1 pg/mL. Changes in serum PTH concentrations were observed across the months with significantly higher levels measured during the winter months versus the other seasons (77.8 pg/mL versus 60.5 pg/mL, *p* < 0.0001, Mann–Whitney test). No other significant linear correlation was found between 25(OH)D, and the other tested factors, in particular, T-score (hip and spine), BMD (hip and spine), or dietary calcium intake.

### 3.3. Factors Associated with Low 25(OH)D Concentrations

Unidimensional analyses were performed to determine which subject characteristics were associated with hypovitaminosis D and to identify the potential risk factors. As the number of subjects with 25(OH)D concentration > 20 ng/mL was insufficient to ensure adequate statistical power, the cutoff value of 17 ng/mL was considered for these analyses. This value corresponded to the 75th percentile for serum 25(OH)D and allowed a less unbalanced distribution of subjects between groups. A low vitamin D status, defined as 25(OH)D concentrations lower than 17 ng/mL, was significantly associated with obesity (BMI > 30 kg/m^2^) (*p*=0.05) (Table 2). There was also a tendency toward an association with veiling (*p* < 0.1).

A stepwise multiple logistic regression model was developed to further explore the independent variables with a statistical or a near-statistical association with low vitamin D status, defined as 25(OH)D levels < 17 ng/mL, in the univariate analysis. Hypovitaminosis D was found to be significantly associated with obesity (BMI in two categories: < 30 kg/m^2^ versus ≥ 30 kg/m^2^; OR = 1.826; 95% CI 1.081, 3.083; *p*=0.02), veiling (yes versus no; OR = 3.526; 95% CI 1.090, 11.400; *p*=0.0353), and the season (summer versus other pooled seasons; OR = 4.159; 95% CI 2.456, 7.043; *p* < 0.0001).

## 4. Discussion

To our knowledge, this is the first report on the vitamin D status of a group of postmenopausal women living in a sunny area of Northern Algeria. Contrary to what would be expected in an area with such abundant sunshine, results show that the prevalence of vitamin D inadequacy is very high. Indeed, more than 85% of subjects have 25(OH)D levels lower than 20 ng/mL, and about 20% of subjects exhibit severe vitamin D deficiency (< 10 ng/mL). These results are in agreement with the few previous studies that report very high prevalence of hypovitaminosis D in comparable populations of North African sunny regions, at roughly the same latitudes (about 30° N) [25–27]. Hence, in a cohort of 429 postmenopausal women recruited in North Morocco, the mean 25(OH)D level was 14.5 ± 12.4 ng/mL and 78.1% had levels < 20 ng/mL [26]. Consistently, in a Tunisian population consisting of 389 subjects, including 61% of females, largely housewives, Meddeb et al. found that 59.5% of the 50–59-year age group had an average 25(OH)D level < 15 ng/mL [27].

Two other major findings are compatible with results generally reported in populations with hypovitaminosis D and further support the validity of our sample set and analysis.

As universally observed, a significant seasonal variation of serum 25(OH)D levels is evident (*p* < 0.0001). This finding is generally explained by the fluctuations of sunshine duration, exposure, and intensity over seasons. Interestingly, there is one major peak throughout the year, globally covering the summer months and roughly corresponding to the maximum insolation period. A comparable profile of seasonal variation of 25(OH)D has recently been observed in a large Mediterranean cohort exposed to a similar climate as that existing in North Algeria [28].

Furthermore, the well-documented inverse relationship between 25(OH)D and PTH is observed. Low levels of 25(OH)D cause secondary hyperparathyroidism, which preserves normocalcemia at the expense of bone health. The 25(OH)D concentration below which high levels of PTH ensue has not been established in this study.

In view of the high prevalence of hypovitaminosis D (25(OH)D levels < 20 ng/mL) in the study population, groups with and without a low vitamin D status are unexpectedly highly unbalanced in size. To gain statistical power and to better identify the factors associated with a low vitamin D status, a univariate analysis was performed using the 75th percentile as the cutoff value, that is 17 ng/mL. With this cutoff level, no correlation is found between PTH and 25(OH)D levels. This is consistent with previous data showing that an increase in PTH is detected with 25(OH)D concentrations equal to or below 15 ng/mL [29–31] or between 10 and 20 ng/mL [2].

No significant independent association has been detected between 25(OH)D levels and BMD at any measured site. This is in agreement with some, but not all studies. Rassouli et al. found that serum 25(OH)D was weakly correlated with the spine but not with the hip BMD in early postmenopausal women with vitamin D deficiency [32]. In the Multiple Outcomes of Raloxifene Evaluation study, a 25(OH)D level below 10 ng/mL was associated with a 4% lower trochanteric BMD, although the lumbar spine or the femoral neck BMD did not differ [33]. More recently, no significant difference in BMD or radius BMD loss was demonstrated among women with 25(OH)D levels below or above 30, 20, or 12 ng/mL [34]. These discordances in the skeletal site BMD correlation with 25(OH)D are likely to be linked to differences in sample sizes, selection criteria, country of origin, and perhaps dietary calcium and vitamin intakes among the studied populations. Nevertheless, Bischoff-Ferrari and coworkers reported a correlation, although low and variable among skeletal sites, between these two parameters [35]. This may also support the concept of multifactorial effects of vitamin D on BMD.

After a multilinear regression analysis, being in any other seasons than summer, being obese, and veiling have been found to be independently associated with a vitamin D status lower than 17 ng/mL. The percentage of unveiled women in our sample is too small (3.9%) to establish a strong and definite association with the vitamin D status. However, the significant influence of the clothing style is not surprising and has already been suggested, notably in Turkey, Middle East countries, and Tunisia [13, 27, 36, 37]. In a study conducted in 251 postmenopausal osteoporotic Lebanese women, 25(OH)D levels were significantly lower in women with a dress code covering their arms compared with those who did not follow any special dress code (15.1 ± 6.6 ng/mL versus 22.5 ± 10.6 ng/mL, *p* < 0.001) [36]. Dress code was identified as an independent predictor of 25(OH)D inadequacy (*p* < 0.001). A similar difference according to the clothing style was observed in 2013 Jordanian women of reproductive age [37]. Prevalence of deficiency was 1.60 times higher for women covered with a scarf/hijab (95% CI: 1.06–2.40, *p*=0.024) compared with unveiled women. These consistent observations confirm the importance of sun exposure in the synthesis of vitamin D.

In this study, obesity (BMI > 30 kg/m^2^) has also been found to be a major predictor of the vitamin D status of postmenopausal women. A large amount of evidence resulting from observational studies supports a significant link between obesity and lower serum levels of 25(OH)D regardless of the age [38–40]. Different mechanisms have been proposed to explain this relationship, such as the decreased sun exposure because of decreased mobility and issues of social acceptance or the negative feedback from elevated 1,25(OH)_2_D and PTH levels on hepatic synthesis of 25(OH)D [39, 41]. However, the sequestration and the volumetric dilution theories are probably the most supported within the literature [39]. Wortsman et al. were the first to provide strong evidence that the fat-soluble vitamin D may become trapped within the adipose tissue [42]. Compared with control lean subjects, obese subjects had a significantly attenuated increase in 25(OH)D concentration after a whole-body exposure to UVB irradiation, despite a greater body surface area. More recently, convincing data suggest that volumetric dilution may simply explain the low vitamin D status in obesity. In a cohort of 686 otherwise healthy individuals (BMI range: 16.5 to 61.2 kg/m^2^), an inverse relationship between serum 25(OH)D and body weight was confirmed but, once adjusted for body size, difference in 25(OH)D levels between nonobese and obese individuals was removed [43]. Consistently, interventional studies with vitamin D supplements have shown that obese patients need higher vitamin D dosages than lean individuals to achieve the same 25(OH)D concentrations [44, 45]. Hence, the recommendation for obese subjects is an intake of two or three times more vitamin D than their age group reference [5]. Nevertheless, whether vitamin D deficiency leads to obesity or obesity is responsible for low 25(OH)D concentrations is still a matter of debate [46–49].

The main limitations of our study lie in its cross-sectional design, in the limited geographical area of the recruitment, and in the recruitment process of the participants, mostly housewives with a low education level. However, although caution should be exercised while generalizing our results, the educational and occupational status of the women from our sample does not substantially deviate from that of the Algerian postmenopausal population. Furthermore, the climatic conditions of Douera (seasonality and UVB radiation) correspond to those found near the North coast of the country where approximately 90% of the Algerian population is living. Another study limitation is the measurement of the 25(OH)D level performed (i) at a single time point, not the same for all subjects which may have created a confounding seasonal effect, and (ii) using a 25(OH)D electrochemiluminescence assay that is now known to underestimate 25(OH)D levels, especially at low levels [26]. Finally, the accuracy of self-reported data concerning lifestyle practices may have been subject to report bias; as with such types of studies, we acknowledge that there may be unrecognized confounding. However, such a high prevalence of hypovitaminosis D determined using other 25(OH)D dosage methods has also been reported in neighboring countries or countries located at te same latitude. Furthermore, the predictive factors of low vitamin D status we identified by analysing our data according to a robust statistical methodology have all been already identified as such in other populations.

In conclusion, the most important finding of our study is the critically high prevalence of hypovitaminosis D observed in postmenopausal otherwise healthy North Algerian women, reaching more than 85%. Although derived from a subpopulation of small sample size, these unexpected results in an abundant sunshine region such as North Algeria are of major concern considering the accumulating evidence that vitamin D deficiency may be linked to a multitude of health risks such as osteoporosis, diabetes, and cardiovascular diseases [50]. Nonsummer season, obesity, and veiling emerged as reliable predictors of hypovitaminosis D. These findings emphasize the need to focus the monitoring of vitamin D status in these specific vulnerable subgroups of Algerian postmenopausal women. Further studies are needed to confirm our findings in a larger representative population of postmenopausal women from different areas of Algeria before to address the best policies to overcome hypovitaminosis D in these at-risk women. However, given the growing burden of obesity in the Algerian population [51], an increase intake of vitamin D-containing foods should be encouraged, and vitamin D supplementation should be proposed more systematically. Calcium supplementation should also be proposed concomitantly given the dramatically low calcium dietary intake observed in most of the postmenopausal women studied and the well-known importance of vitamin D and calcium status adequacy for bone health at menopause [4].

## Figures and Tables

**Table 1:** Sociodemographic, biochemical, and clinical characteristics of the study population (*n* = 336).

		Mean ± SD or percentage	Min-max
*Sociodemographic parameters*			
Age (years)	—	60.1 ± 8.5	45.0–87.0
Age at menopause (years)	—	47.4 ± 4.8	26.0–57.0
Seasons of recruitment	Winter	23.5%	—
	Spring	26.8%	—
	Summer	25.0%	—
	Autumn	24.7%	—
Number of children	—	6.5 ± 3.5	0.0–16.0
Education level/schooling (%)	None	57.7%	—
	Primary education	24.4%	—
	Average level	15.5%	—
	University level	2.4%	—
Skin phototype	I–III	50.6%	—
	IV	43.8%	—
	V	5.7%	—
Veiling	Yes	96.1%	—
	No	3.9%	—
Rural or urban area	Urban	74.1%	—
	Rural	25.9%	—
Daily calcium intake	—	491.0 ± 181.3	149.6–1334.9
	<500 mg	58.3%	—
	≥500–<700 mg	30.1%	—
	≥700–<800 mg	5.4%	—
	≥800 mg	6.3%	—
Daily vitamin D intake (IU)	—	56 ± 44	0–220
*Clinical parameters*			
BMI (kg/m^2^)	—	28.5 ± 5.3	15.0–50.0
T-score ≤ −2.5	Hip	12.8%	—
	Lumbar spine	32.2%	—
Osteoporosis^∗^	—	32.8%	—
History of fracture	Yes	16.1%	—
*Biochemical parameters*			
25(OH)D (ng/mL)	—	14.4 ± 5.3	4.0–35.9
PTH (pg/mL)	—	64.2 ± 25.1	14.0–179.6

^∗^T-score of the lumbar spine and/or lower hip ≤ −2.5; IU: international units; SD: standard deviation.

**Table 2:** Results of the univariate and multivariate logistic regression models for the association of subject characteristics with vitamin D hypovitaminosis with the cutoff value of 17 ng/mL^∗^.

Characteristic	*p* value^∗^	Modalities for OR calculation	OR (95% CI)
*Univariate logistic regression*			
Age	0.41	Continuous	0.99 (0.96–1.02)
Duration of menopause	0.46	Continuous	0.99 (0.97–1.02)
Parity	0.75	Continuous	1.01 (0.94–1.09)
BMI	0.05	<30 kg/m^2^ versus ≥30 kg/m^2^	0.61 (0.36–1.01)
Habitat/housing	0.32	Urban versus rural	1.31 (0.76–2.26)
Education level	0.81	Education versus no education	0.82 (0.50–1.33)
Skin phototype	0.82	Dark versus light	1.13 (0.69–1.84)
Veiling	0.09	Yes or no	2.56 (0.84–7.84)
T-score spine	0.95	Continuous	0.99 (0.83–1.18)
DMO spine	0.93	Abnormal versus normal	0.98 (0.58–1.65)
T-score hip	0.19	Continuous	1.12 (0.93–1.36)
DMO hip	0.67	Abnormal versus normal	0.80 (0.49–1.31)
*Multivariate logistic regression*			
BMI	0.02	<30 kg/m^2^ versus ≥30 kg/m^2^	1.826 (1.081–3.083)
Veiling	0.0353	Yes or no	3.526 (1.090–11.400)
Season	<0.0001	Summer versus other pooled seasons	4.159 (2.456–7.043)

^∗^ *p* value was calculated for each variable according to the Mann–Whitney test or chi-square test.
